# Supplementary material for: Employment of Phenolic Compounds from Olive Vegetation Water in Broiler Chickens: Effects on Gut Microbiota and on the Shelf Life of Breast Fillets
Source: Molecules. 2021 Jul 16;26(14):4307. doi: 10.3390/molecules26144307 (PMC8306377; doi:10.3390/molecules26144307)
Supplement: Supplementary file 1 [file molecules-26-04307-s001.zip › molecules-1290809-supplementary.pdf]

Table S1. Nutrient composition of experimental diets.

	Starter diet<br>0-23 d	Grower diet<br>24-37 d	Finisher diet<br>38-48 d
Crude protein, %	20.1	18.9	17.0
Ether extract, %	5.05	4.93	5.37
Crude fiber, %	1.19	1.19	1.34
Ash, %	5.19	5.50	5.26
Lysine, %	1.29	1.20	1.13
Metionine, %	0.31	0.28	0.31
Vitamin–mineral mix supplied the following per kilogram of diet			
Vitamin A, UI	11,880	11,880	9,720
Vitamin D3, UI	4,400	4,400	3,600
Vitamin E, UI	53.0	53.0	43.0
Phytase, FTU	500	500	500
Canthaxanthin, mg	2.00	2.00	4.00
Lutein, mg	16.0	17.2	60.8
Zeaxanthin, mg	1.00	1.70	3.80
Copper sulphate pentahydrate, mg	52.0	52.0	42.0
Sodium selenite, mg	0.46	0.46	0.38
Calcium iodate anhydrous, mg	1.71	1.71	1.40
Iron (II) carbonate, mg	73.0	73.0	60.0
Zinc oxide, mg	109	109	89.0
Manganous oxide, mg	118	118	89.0
Maduramycin ammonium, mg	5.00	5.00	-

Diets were produced by a commercial feed mill (Martini, Budrio di Longiano, Italy).

Table S2. Phenols concentration in the Crude Phenolic Concentrate (CPC) and in the diets supplied to the broilers from 24 to 48 days. Values are mean±standard deviation measured the day of diet preparation.

Phenolic compound	CPC (g/L)*	Diet L0 (mg/kg)	Diet L1 (mg/kg)**	Diet L2 (mg/kg)**
3,4-DHPEA	3.2±0.2	<LOD	97.0±1.24	174.6±0.85
p-HPEA	0.6±0.0	<LOD	8.7±0.04	13.0±0.42
Verbascoside	0.7±0.1	<LOD	5.88±0.03	24.8±0.21
3,4-DHPEA-EDA	10.1±0.1	<LOD	63.9±0.41	107.7±1.11
Sum of phenols	14.6±0.3	<LOD	175.5±1.3	320.2±1.5

*Actual concentration of phenols in the CPC (liquid form) at the moment of use; **actual concentration of phenols into the diet measured the same day of feed preparation; 3,4-DHPEA: Hydroxytyrosol; *p*-HPEA: Tyrosol; 3,4-DHPEA-EDA: dialdehydic form of the decarboxymethyloleuropein aglycone; LOD: at 278 nm 3,4-DHPEA 100 µg/kg; *p*-HPEA 90 µg/kg; Verbascoside 295 µg/kg; 3,4-DHPEA-EDA 285 µg/kg.

Table S3. Microbial targets (CFU/g) evaluated along the shelf life of chicken breast samples.

Time	Diet	TVC	*Pseudomonas*	*Shewanella* spp.	*Enterobacteriaceae*	LAB	TPC
T24	L0	3.54	2.00	2.00	2.80	3.71	1.99
	L1	3.75	2.15	2.00	2.85	4.16	2.13
	L2	3.35	2.00	2.00	3.00	3.83	1.56
	P	ns	ns	ns	ns	ns	ns
T72	L0	3.97	2.65	2.29	3.60	3.93	2.39
	L1	3.22	2.73	2.30	3.14	3.70	2.41
	L2	3.92	2.67	2.94	3.65	3.76	2.81
	P	ns	ns	ns	ns	ns	ns
T120	L0	4.24	3.84	2.69	3.46	4.21	3.37
	L1	4.34	3.84	2.61	3.66	4.04	3.21
	L2	4.14	3.58	3.27	3.25	4.04	3.59
	P	ns	ns	ns	ns	ns	ns
T168	L0	4.27	4.36	3.27	2.78	3.83	3.68
	L1	5.33	5.15	4.15	3.82	4.26	4.80
	L2	4.72	4.58	3.50	3.40	3.88	4.48
	P	ns	ns	ns	ns	ns	ns
T216	L0	5.91	6.85	4.62	4.65	4.31	5.84
	L1	6.48	6.92	4.43	5.33	5.20	7.06
	L2	7.15	7.07	4.91	4.04	5.24	7.18
	P	*	ns	ns	**	ns	ns
T264	L0	7.36	6.80	5.87	5.53	5.23	7.57
	L1	7.82	7.77	4.81	5.77	5.53	7.99
	L2	7.90	8.15	6.77	6.67	6.66	8.29
	P	ns	**	**	ns	ns	ns

TVC: total viable count; TPC: total psychrotrophic count; LAB: Lactic acid bacteria. P represented the Partial P values of the NPC test; ns: p >0.05; *: p <0.05; **: p <0.01.

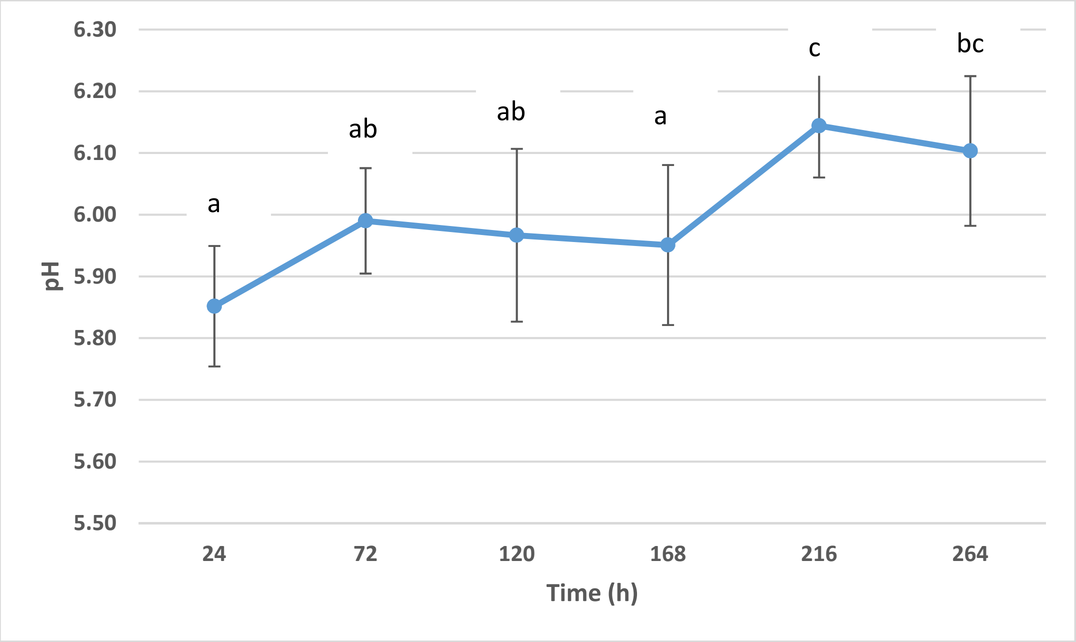

Figure S1. Effect of time on pH of raw chicken meat.

All data points represent mean and their standard deviations. Means with no common superscript differ significantly ($p < 0.05$).

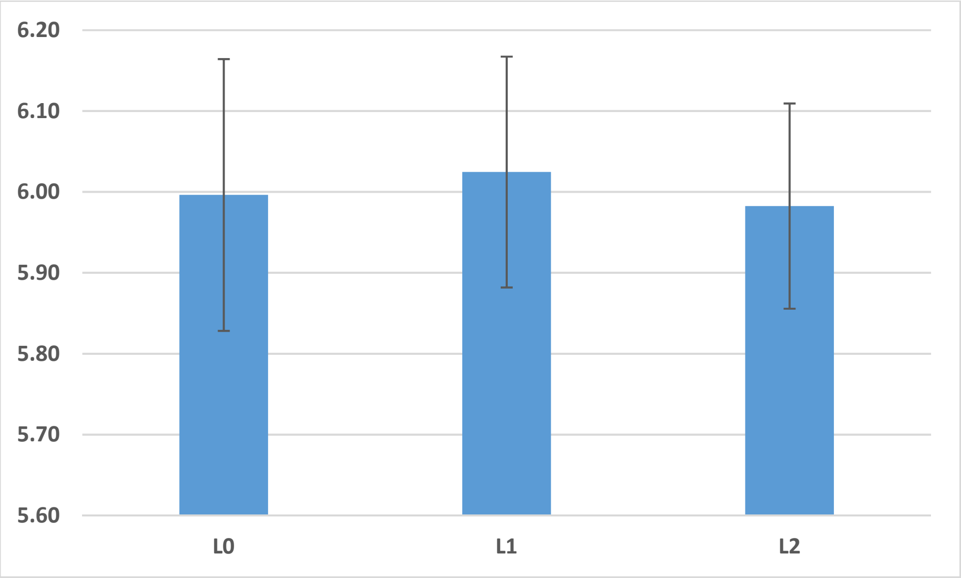

Figure S2. Effect of diet treatment on pH of raw chicken meat.

All data points represent mean and their standard deviations.

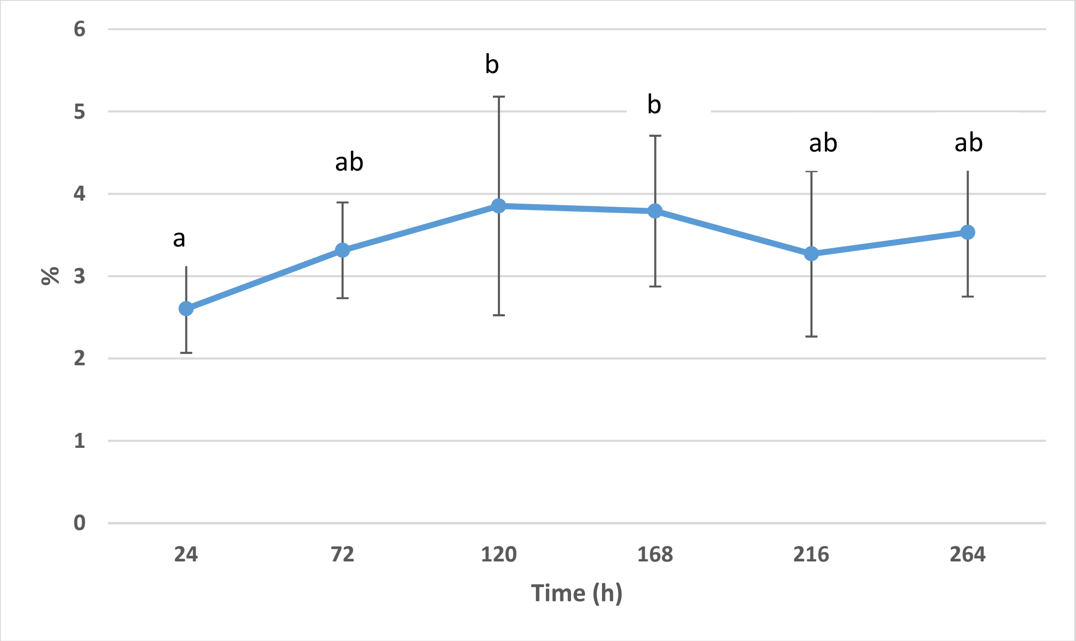

Figure S3. Effect of time on drip loss (%) of raw chicken meat.

All data points represent mean and their standard deviations. Means with no common superscript differ significantly ($p < 0.05$).

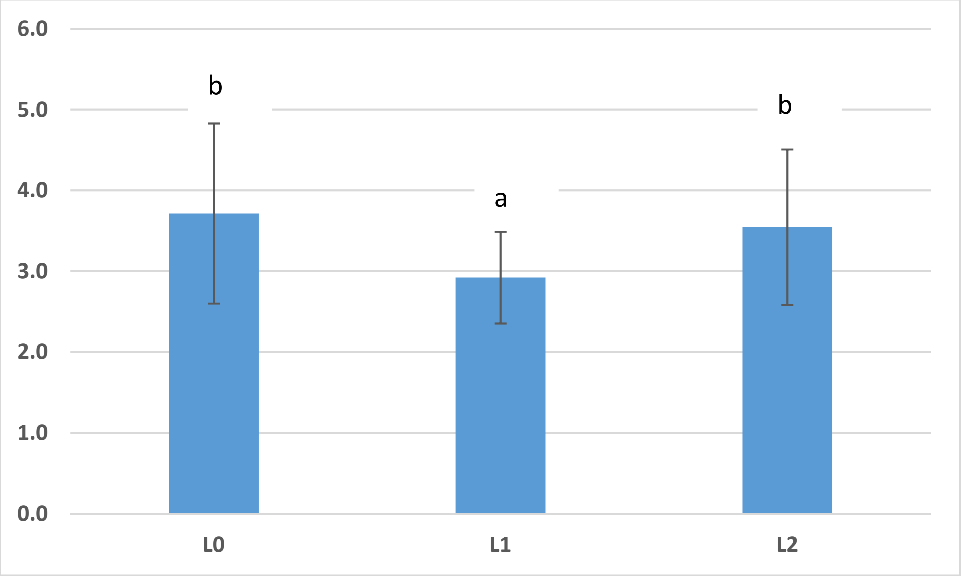

Figure S4. Effect of diet treatment on drip loss (%) of raw chicken meat.

All data points represent mean and their standard deviations. Means with no common superscript differ significantly ($p < 0.05$).

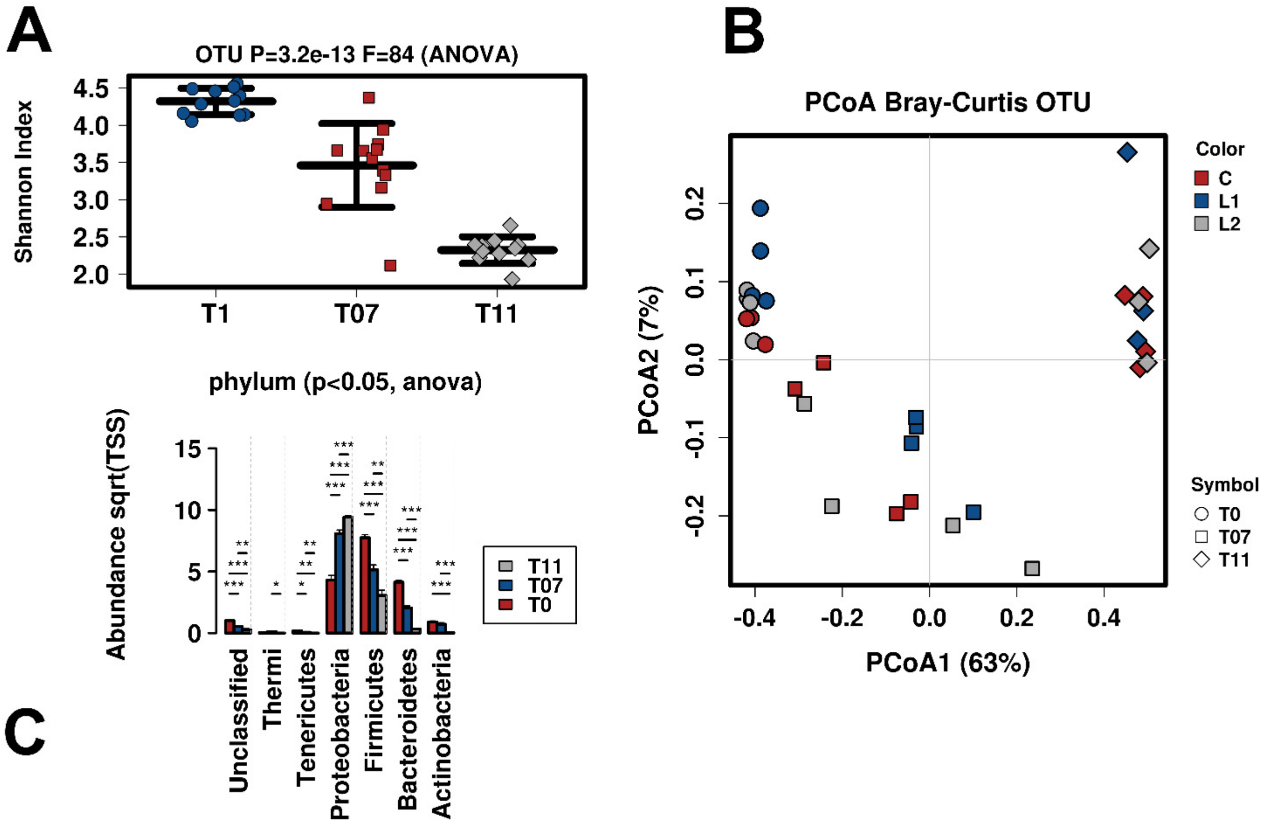

Figure S5. The modification of the biodiversity (A) the composition of the community in PCoA (B) and phyla predominance (C) during shelf life of breast meat at 24 (T1), 168 (T07) and 264 (T11) hours.
